# A Review of the Potential Consequences of Pearl Millet (*Pennisetum glaucum*) for Diabetes Mellitus and Other Biomedical Applications

**DOI:** 10.3390/nu14142932

**Published:** 2022-07-18

**Authors:** JinJin Pei, Vidhya Rekha Umapathy, Srinivasan Vengadassalapathy, Shazia Fathima Jaffer Hussain, Ponnulakshmi Rajagopal, Selvaraj Jayaraman, Vishnu Priya Veeraraghavan, Chella Perumal Palanisamy, Krishnasamy Gopinath

**Affiliations:** 1 Qinba State Key Laboratory of Biological Resources and Ecological Environment, 2011 QinLing-Bashan Mountains Bioresources Comprehensive Development C. I. C, Shaanxi Province Key Laboratory of Bio-Resources, College of Bioscience and Bioengineering, Shaanxi University of Technology, Hanzhong 723001, China; xnjinjinpe@163.com; 2 Department of Public Health Dentistry, Sree Balaji Dental College and Hospital, Pallikaranai, Chennai 600100, India; drvidhyarekha@gmail.com; 3 Department of Pharmacology, Saveetha Medical College and Hospital, Saveetha Institute of Medical & Technical Sciences, Chennai 602105, India; srinivasanv.smc@saveetha.com; 4 Department of Oral and Maxillofacial Pathology, Ragas Dental College and Hospitals, Chennai 600119, India; shaziafathimarizwan@gmail.com; 5 Central Research Laboratory, Meenakshi Academy of Higher Education and Research (Deemed to be University), Chennai 600078, India; drponnulakshmi.researchscientist@madch.edu.in; 6 Centre of Molecular Medicine and Diagnostics (COMManD), Department of Biochemistry, Saveetha Dental College & Hospital, Saveetha Institute of Medical & Technical Sciences, Saveetha University, Chennai 600077, India; vishnupriya@saveetha.com; 7 State Key Laboratory of Biobased Materials and Green Paper Making, School of Food Science and Engineering, Qilu University of Technology, Shandong Academy of Sciences, Jinan 250316, China; 8 Faculty of Medicine, Integrative Physiology and Pharmacology, Institute of Biomedicine, University of Turku, 20014 Turku, Finland

**Keywords:** diabetes mellitus, pearl millet, nutritional importance, health effects

## Abstract

Diabetes mellitus has become a troublesome and increasingly widespread condition. Treatment strategies for diabetes prevention in high-risk as well as in affected individuals are largely attributed to improvements in lifestyle and dietary control. Therefore, it is important to understand the nutritional factors to be used in dietary intervention. A decreased risk of diabetes is associated with daily intake of millet-based foods. Pearl millet is a highly nutritious grain, nutritionally comparable and even superior in calories, protein, vitamins, and minerals to other large cereals, although its intake is confined to lower income segments of society. Pearl millet contains phenolic compounds which possess antidiabetic activity. Thus, it can be used to prepare a variety of food products for diabetes mellitus. Moreover, it also has many health benefits, including combating diabetes mellitus, cancer, cardiovascular conditions, decreasing tumour occurrence, lowering blood pressure, heart disease risk, cholesterol, and fat absorption rate. Therefore, the current review addresses the role of pearl millet in managing diabetes.

## 1. Introduction

Diabetes mellitus is also known as diabetes, a group of metabolic disorders which are characterized by high blood sugar level (hyperglycemia) over a prolonged period of time. [1]. Globally, the occurrence of diabetes is projected to elevate from 2.8% (in 2000) to 4.4% (in 2030). It might be projected that the number of diabetic patients will increase to over 366 million cases in 2030 [2]. It has been well understood that a diabetic patient’s glucose level rises exponentially beyond the usual range after a meal. It is also true that the amount of their blood glucose quickly decreases when the body struggles to retain the extra glucose for future use. Types 1 and 2 are classified as diabetes. Type 1 diabetes is also defined as juvenile diabetes or insulin-dependent diabetes as a patient’s pancreas is unable to manufacture or produce insulin [3]. However, type 2 diabetes (T2D) usually occurs first in adults whenever the body becomes insulin resistant or fails to deliver enough insulin [4]. T2D accounts for 90% of people with diabetes worldwide. It can mainly be the result of physical inactivity and being overweight. The complication added to T2D is that it has less symptoms reported than type 1 diabetes and is often only diagnosed when side effects have already occurred [5].

Atherosclerosis, which makes the blood vessels hard and narrow, includes major complications caused by hyperglycemia. Heart disease, stroke, retinopathy, and kidney failure are some other diabetes-related health issues [6,7]. Diabetic retinopathy leads to blindness by cumulatively weakening the tiny blood vessels in the eye, leading to 1% of cases of blindness worldwide. Likewise, a very common complication is kidney failure due to constant restricted blood flow. Increased blood glucose could also cause nerve damage that may lead to the need for amputation of the limb. These disorders decrease the standard of living of the patients and potentially their interaction with others [8].

Increased bone fracture risks in both Type 1 and 2 diabetics are also additional complications [9,10,11]. It is, however, noteworthy that Types 1 and 2 diabetic individuals have lower and higher mineral densities than stable individuals, while all risk of fractures is high. Other diabetes-related causes may clarify fracturing threats associated with various bone mineral densities. T2D individuals also have a higher body mass index and little physical activity, meaning that every fall is more likely to be fractured [12,13]. The rise in the body’s glucose level physiologically interrupts with glycation, which consequently lowers collagen cross-linking and, despite the higher bone mineral density, results in more brittle bone [10,14]. In addition, lower bone turnover rates in diabetic patients cause poor fracture healing by interfering with alternate glycaemia with a key bone remodelling regulator, the parathyroid hormone [15]. T2D therefore contributes to a higher fracture risk in many convergent ways. Consequently, fractures further limit patients’ mobility, making diabetes worse.

Muscle fatigue due to poor glycaemic control is another symptom that a T2D patient would have to tolerate [16]. In turn, this causes fatigue and lack of energy, leading to demotivate patients from participating in physical exercise. Also, as the body derives energy from muscle breakdown, patients lose muscle mass. Such abnormal muscle anabolism makes reduction of muscle mass one of the main risks that a patient with diabetes has to face [17,18]. The consequent loss of motor function gives the patient’s additional physical as well as psychological complications.

## 2. Millets for Diabetes Control

Diary treatments are a simple and economical way to deliver preventive benefits and enhance their quality of life for those living with type-2 diabetes [19]. Hence, the current recommendations for type-2 diabetes are to adopt safe, nutritious diets, particularly with low-GI (glycaemic index) starchy carbohydrates and elevated dietary fibre that can help control post-prandial hyperglycaemia and minimize body weight. Low glycaemic carbohydrate/high fibre diet has been shown to successfully lower plasma cholesterol and enhance blood glucose balance for type 2 diabetes [20].

**Figure 1:**
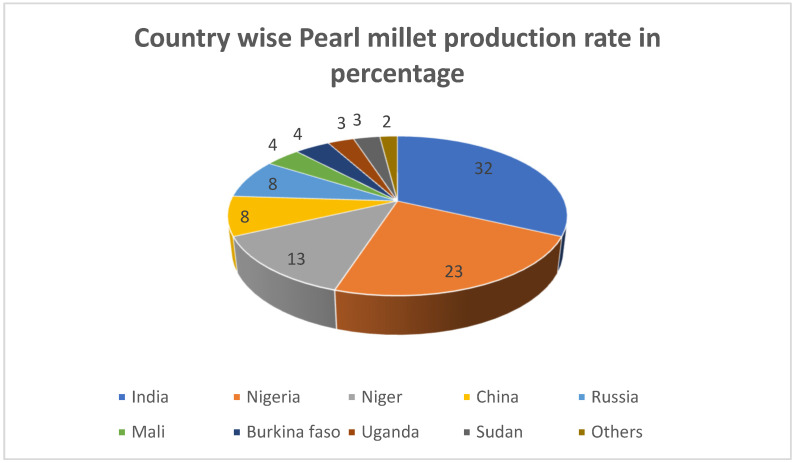
Pearl millet production rate (in percentages) of different countries.

Millet is an important crop for African, Asian, and European populations. Nutritionally, it is superior to other significant cereals such as wheat and rice. Moreover, it is an attractive ingredient for the introduction into balanced foods because it has many vital nutrients [21]. In African and Asian regions, millet grains have been used efficiently to produce adult meals, drinks, and weaning foods such as porridge, bread (fermented and unfermented), and snacks, which form the principal component of traditional foods [22]. Figure 1 shows the millet production rate of various countries in the world [23]. Recent studies have analysed the positive implications of millet on type 2 diabetes risk markers [24]. However, the results of randomized trials to evaluate the GR (glycaemic response) effects of millet were contradictory, with some using brief intervention times or limited samples that would minimize the validity of the results. Millets have essentially lower amino acid levels and greater fat content. However, 75% of this fat is not detrimental to the heart, and it is safe. It contains polyunsaturated fatty acid, which is highly healthy [25]. Millets such as jowar, ragi, and bajra are used to make a very important part of the Indian diet. Thus, they are also recommended by diabetologists simply because they are recognized for promoting diabetes control steps. The high fibre content of millet allows the level of sugar in the blood stream to slow down. Indeed, it would be more fitting to argue that digestion delays result in a more even distribution of sugar [26]. A diabetic should hope to remain clear from the dangerous increases in blood sugar levels, which cause various complications, by taking millets daily. Diabetologists recommend millets mostly for patients because of their ability to lower the risk of type 2 diabetes and cardiovascular disorders.

## 3. Pearl Millet and Its Nutritional Significance

**Figure 2:**
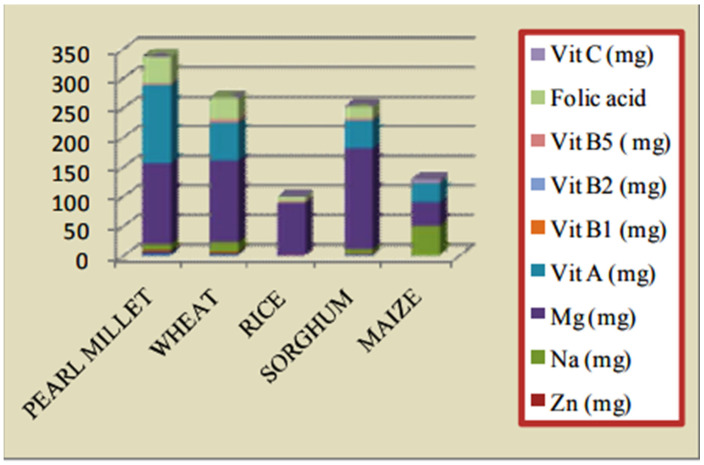
Comparison of nutritional values of pearl millet with other millets.

Pearl millet (*Pennisstum glaucum*) is a multifunctional cereal crop which belongs to the Poaceae family. It is generally referred to in various local Indian languages as bajra, bajri, sajje, kambu, kamban, sajjalu, etc. It is widely used for food and forages [27]. Pearl millet is the third largest major crop after rice and wheat in India. It was developed on an area of 7.4 million, averaging 9.13 million tons, in 2017–2018 [28]. Rajasthan, Maharashtra, Gujarat, Uttar Pradesh and Haryana are the largest pearl millet growing states in the country [29]. The higher nutrient content means that pearl millet has been recognized by the Ministry of Agriculture, Government of India as one millet under “Nutri-Cereals” (GOI). Pearl millet has a higher digestibility of fat than most cereals. It is also rich in unsaturated fatty acids with higher nutrient omega-3 fatty acid content (Figure 2). Pearl millet has a maximum content of macronutrients and is considerably rich in resistant starch and soluble and insoluble dietary fibre in contrast to other millets [30]. Basically, pearl millet has a large root structure, which absorbs soil nutrients and has a higher importance for nutrition than other cereal crops, including wheat, rice, maize, and sorghum. A high degree of iron, zinc, magnesium, copper, manganese, potassium, and phosphorus is found in the mineral. This is a strong energy source with a calorific value of 361 Kcal/100 g and a high amount of fibre (1.2 g/100 g) [29]. It is higher [31] and is a healthy source for vitamin B, vitamin A, folic acid, calcium, and magnesium [32]. Pearl millet grain has a higher fat content than other cereals which causes low product quality [33]. Table 1 indicates the nutritional values of pearl millet [34].

The starch content in various pearl millet genotypes ranges from 62.8 and 70.5%, soluble sugar between 1.2 and 2.6%, and amylose between 21.9 and 28.8% [35]. In some high-yielding Indian pearl millets, lower values for starch (56.3 to 63.7%) and amylose (18.3 to 24.6%) were found [36]. The key portion of overall soluble sugar (2.16 to 2.78%) was sucrose (66%), followed by raffinose (28%) [37]. Stachyose, glucose, and fructose were other sugars found in measurable quantities. The percentage of overall sucrose in pearl millet was smaller than sorghum. Pearl millet, like sorghum, is normally 9% to 13% protein, but significant changes in protein, 6% to 21%, were observed [38]. Lysine is the pearl millet protein’s first minimal amino acid. There is a strong inverse association between grain protein level and protein lysine content [39]. Significant inverse associations were also reported between protein and threonine, methionine, and tryptophan in high-protein varieties of pearl millet with a protein content between 14.4 and 27.1%. The essential amino acid profile contains more lysine, threonine, methionine, and cystine (Table 1) in pearl millet protein than in sorghum and other millet proteins [40]. Its contents of tryptophan are also higher. There have been variations in lipid extraction methods and genetic heterogeneity that led to differences in pearl millet fatty acids [41]. Linoleic, oleic, and palmitic acids were the major fatty acids, both free and bound. In neutral lipids, phospholipids, and glycolipid fractions, differences in composition of fatty acid were noted [42]. Linoleic and palmitic acid is the highest neutral lipid; oleic acid phospholipid was the lowest. The total dietary fibre of pearl millet (20.4%) and finger millet (18.6%) are higher than sorghum (14.2%), wheat (17.2%), and rice (8.3%), and the total dietary fibre content of pearl millet was 17% [43].

## 4. Pearl Millet and Diabetes

Pearl millet helps to keep blood sugar levels stable for a long time in diabetic patients. It is also helpful for diabetes patients because it has a comparatively small glycaemic index that helps steadily digest and contain glucose at a slower pace than other foods [44]. This will help healthy blood sugar levels for long stretches.

The amylase activity of pearl millet is very high, about 10 times than that of wheat. Maltose and D-ribose are the major sugars in the flour, and are low in fructose and glucose [45]. Diet is known as the centrepiece of diabetes mellitus treatment, especially important in the case of non-insulin-dependent diabetes mellitus (NIDDM), which involves the metabolism of glucose and secondary lipid and protein deficiencies as the primary derangement [46]. Diabetes dietary treatment includes reducing postprandial hyperglycaemia and strong glycaemic control. The Glycaemic Index (GI) definition originated as a physiological basis for the classification of carbohydrate foods based on the blood glycosis reaction which they consume, and was introduced by Jenkins et al. (1981) [47]. Mani et al. (1993) stated that pearl millet (*Penniseteum typhoideum*) is the lowest GI compared to varagu alone in addition to complete green grams (Phaseolus aureus Roxb), jowar (Sorghum vulgare), and ragi (Eleusine coracana) [48]. Low-glycaemic foods are beneficial for enhancing the metabolic regulation of blood pressure and low-density plasma lipo protein cholesterol leading to less prominent insulin reactions [49]. Several new food items based on pearl millet can be created, and conventional recipes for diabetic patients need to be supported.

It has also been shown that millet-based foods (pearl, foxtail, and finger) have been correlated with low GIs in both stable and type 2 diabetes because of their high protein level [50]. Shukla et al. (1991) found that the GR of bajra chapati was significantly lower in stable individuals than white bread. In addition, adding 30 g of fenugreek to millet chapati further decreased GI (Glycaemic Index), which resulted in less GR than that observed by the ingestion of fenugreek millet chapati. In this situation, the GR (Glycaemic Response) reduction could have been due to the quality and viscosity of the fenugreek fibre on the leaves, which may slow GE [51]. The positive relation between the proso millet intake in type 2 diabetic participants and a substantial reduction in the glucose effect has been well founded [52]. Colling et al. (1981) note that glycaemic and insulinemic responses may be influenced by the process and time taken to prepare a meal [53]. The degree of frying and the length of fermentation influenced these findings in particular.

Sukar et al. (2020) [54] showed substantial elevation of adiponectin associated with a vast decrease in blood glucose levels during the study periods. These findings imply that, feeding with the whole grain of pearl millet, a diet can play a significant role in restoring the plasma level of adiponectin to the physiological level. It is well established that an increase in adiponectin level stimulates glucose utilization through the activation of AMP-activated protein kinase in the skeletal muscle and liver [55], and such a diet containing pearl millet could reduce glucose level due to an enhancement of the utilization of glucose by peripheral tissues and the elevation of adiponectin levels. Many theories support the hypoglycaemic effects of pearl millet, such as the theory that pearl millet being rich in phytate and phenolic compounds reduces fasting hyperglycaemia and an attenuated postprandial blood glucose response in rats. Phenolic compounds are also known to enhance insulin activity, and pearl millet regulates intestinal GLUT, increases muscle glucose uptake, and reduces hepatic gluconeogenesis [56].

Cereal grains, especially pearl millet, are rich in antioxidant properties and bioactive compounds, as well as other important minerals. Extracts from pearl millet are reported to offer protection against DNA damage. Developing a method that can improve the nutritional profile of the natural substrate is of the utmost importance. Various researchers are using biotechnological methods for the improvement/enhancement of the bioactive compounds of cereal grains. One of the successful methods used by scientists/researchers is fermentation technology, which can manifoldly enhance the nutrients of cereal grains. Pearl millet grains are attracting attention because of the presence of certain specific bioactive constituents, their importance for health, and high nutritional values. Generally pearl millet is classified as a low-glycaemic index (GI) food because of its high fibre content. The GI assesses how much the carbohydrate content of food influences the rate and extent of change in post-prandial blood glucose concentration. Apparently, pearl millet, as a low-GI food, helps lower blood glucose available for triacylglycerol synthesis. Besides, millets condense VLDL cholesterol, a carrier of triacylglycerol in plasma, lowering triacylglycerol levels even further. As a result, the consumption of millet grains may play an important role in lowering the level of blood lipids [57].

Prediabetes is a state of elevated plasma glucose in which the threshold for diabetes has not yet been reached and can be predispose to the development of type 2 diabetes and cardiovascular diseases. Insulin resistance and impaired beta-cell function are often already present in prediabetes. Hyperglycaemia can upregulate markers of chronic inflammation and contribute to increased reactive oxygen species (ROS) generation, which ultimately cause vascular dysfunction. Conversely, increased oxidative stress and inflammation can lead to insulin resistance and impaired insulin secretion. Thus, the inhibition of ROS overproduction is crucial for delaying the onset of diabetes and for the prevention of cardiovascular complications. Many kinds of bioactive compounds—such as polyphenols, most flavonoids, and phenolic acids—naturally occur in millet, which might offer various health benefits, as seen in their antioxidant and anti-inflammatory properties [58].

The close correlation between millet consumption and decreased insulin response has already been confirmed. Shukla et al. (1991) found no major variations in IR in stable and type 2 diabetic individuals after the ingestion of pearl millet, while white bread developed somewhat less of an insulin response in type 2 diabetics 1 h after treatment. In stable people, pearl millet demonstrated low GIs and a high insulinemic index; however, the same was true for those with type 2 diabetes with high GIs and a low insulinemic index. The authors observed that pearl millet evoked insulin separation in healthy persons, which decreased the gastrointestinal tract, whereas the insulin reserve in type 2 diabetics could have been inadequate to mobilize insulin after ingestion of pearl millet. Pearl millet is known for its valuable health benefits, primarily due to its high content of polyphenols, which have antioxidant properties [59].

Epidemiological reports have shown that millet eating communities suffer from a lower incidence of diabetes [60]. Pearl millet grains have many functional properties owing to their high fibre content, fatty acid composition, and plant chemicals [61]. The gained understanding of the nutritional effects of pearl millet is of considerable significance in nutritional programmes. Diabetes may usually be caused by hereditary predispositions, obesity, and a heavy intake of high-glycaemic foods. Nani et al. (2015) measured the impact of pearl millet intake on diabetic rat glucose metabolism. The authors suggested that eating pearl millet-based meals could be helpful in fixing type 2 diabetes with induced hyperglycaemia, thereby reducing the severity of the condition, as an alternative to prevention [62]. Hegde et al., (2005) have found that food animals with 55% kodo millet food have decreased hyperglycaemia by 42%, cholesterol by 27%, and non-enzymatic antioxidants (Glutathione, vitamin E, and C) and enzymatic levels by 27% (glutathione reductase) [63]. Millet grains have a greater slow digestible starch quality than some other cereals due to the characteristics of starch, including amylase content, granular structures (polygonal size with porous surfaces), fatty acid volumes, and types (oleic acid content) capable of forming complications with starch molecules and lipid inter-acid starch protein [64]. In addition, the existence of phytochemicals (phenolic acids, flavonoids, and phytats) can lead to inhibiting the activity in monosaecharides of gastrointestinal α-amylase (pancreatics) and α-glycosidase (intestinal) enzymes, reducing the body’s hyperglycaemic presence [65]. However, the method of processing applied to millets will greatly influence the hypoglycaemic character, so it is important to promote the implementation of processes that sustain low starch hydrolysis [66]. Relative to other cereal products, pearl millet produces high amounts of leucine amino acid, inducing insulin secretion through down regulation of adrenergic alpha 2A receptor surface expression via the mammalian target of rapamycin (mTOR) pathways (Leucine secretion pathway). These features are the preferred grains of pearl millet for the treatment of insulin and cardiovascular problems in type 2 diabetes [67].

Some in vivo experiments were performed to research the effect of pearl millet grains on diabetes. In one research, the impact on glucose and insulin responses in diabetic people was assessed in six typical Sudanese carbohydrate-rich meals. A slightly lower response to postprandial glucose and insulin was shown for pearl millet acid (porridge) followed by wheat gorasa (pancakes), while maize acid triggered a higher postprandial glucose and insulin response [68]. Another study showed substantially decreased levels of non-enzymatic antioxidants (glutathione, vitamin E, and vitamin C), enzymatic antioxidants (superoxide dismutase, catalase, glutathione peroxidase, and glutathione reductase), and lipid peroxides of diabetes in normal amounts compared to pearl millet-fed populations [62]. Therefore, pearl millet is also very effective in diabetes management. It gradually digests and contributes glucose to the blood at a higher rate relative to other foods due to its high fibre content. This helps to maintain a steady blood sugar level in diabetic patients for a long time.

## 5. Pearl Millet in the Human Disease Management System

**Figure 3:**
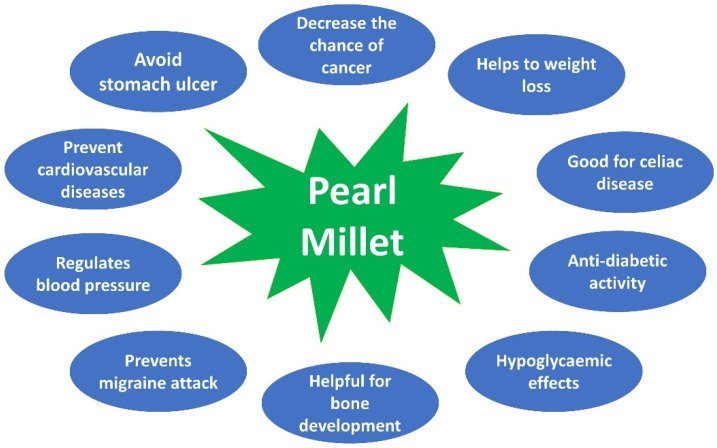
General Biomedical Application of Pearl Millet.

Pearl millet has many nutritional benefits as a result of its rich structure of minerals and proteins. It has high protein content, and it comprises several significant minerals such as magnesium, phosphorus, zinc, etc. It also provides vital amino acids and vitamins that add to a variety of human treatments (Figure 3) [64].

Excess acidity in the stomach following food consumption is the most important explanation for stomach ulcers [69]. Generally, pearl millet is suggested for stomach ulcer treatment, because it is one of the very few grains that alkalizes the stomach and prevents stomach ulcers or decreases the effect of ulcers [70]. Lignin and phytonutrients are good antioxidants in pearl millets that prevent cardiovascular diseases [71]. Pearl millet is also considered healthy for heart protection. There have been high levels of magnesium present in pearl millet, which regulates blood pressure and alleviates heart stress [72]. It has rich magnesium that decreases the incidence of respiratory symptoms in asthma patients and is also helpful in preventing migraine attacks [73]. Pearl millet has high phosphorus content which is very important for bone growth and development as well as for the production of ATP, the body’s energy currency [34]. As millets are known to reduce the risk of cancer, it is expected that pearl millet will have the same effect potentially due to its high content in magnesium and phylate compound [74].

The greatest obstacle facing people who wish to lose weight is to regulate their consumption of calories. Pearl millet will support the weight loss process because the fibre content is high. It takes longer for the grain to travel from the stomach to the intestines, due to the fibre content. This means the pearl millet satiates hunger for a long time and therefore helps to limit the total intake of food [75]. Celiac disease is a disorder in which a person could not endure even a little gluten in the diet. Since millet is gluten-free, it is great for people with celiac disease [76]. Pearl millet is widely recommended for people with elevated cholesterol levels. It comprises a phytochemical known as phytic acid that is estimated to influence the metabolism of cholesterol and balance the cholesterol in the body [77]. Amino acids are important to our body’s smooth activity [77]. Pearl millet is among the few foods that contain all the amino acids that are essential. Sadly, much of these amino acids are destroyed during the cooking process, as they cannot survive high temperatures because of their hypo-allergic properties. It is easier to eat these amino acids in a low cooked form in order to retain as many as possible [78]. It is also recognized that the high fibre content in pearl millet decreases the likelihood of bile incidence. The insoluble fibre content in pearl millet decreases our system’s production of excess bile. Excessive bile secretion of our intestines also worsens the state of gallstones [79]. Pearl millet is safe to use in the diets of babies, lactating women, the elderly, and the convalescent [23].

## 6. Conclusions

Increased nutritional knowledge challenges the food industry to create new food items with distinctive qualities that can improve people’s health. Recent studies highlighted that the development of health-promoting ingredients and functional foods can prevent and control diabetes and other chronic diseases. This review has shown that pearl millet has a significant impact on diabetic individuals. It is a good source of vitamins and minerals, and is very beneficial for diabetic patients. A variety of bioactive compounds present in pearl millet possess numerous health benefits such as antimicrobial, antioxidant, antidiabetic, and hypocholesterolemic effects, as well as hypoglycaemic activity and guarding against diet-related diseases. It is still mostly restricted to household-level communities in rural areas. One important feature of medicinal dietary change and the encouragement of the use of pearl millet may be to include more nutritious and conventional whole-grain and multigrain alternatives for processed carbohydrates. In order to increase the consumption of pearl millet and to take advantage of its immense nutritious potential, diversification of food production and consumption, in tandem with increasing yields, must be promoted at both national and household levels.

## Figures and Tables

**Table 1:** Nutritional value of Pearl Millet.

Nutrients		Amount
Basic Components	Proteins	22 g
	Water	17.3 g
	Ash	6.5 g
Calories	Total Calories	756 cal
	Calories from Carbohydrates	600 cal
	Calories from Fates	71 cal
	Calories from Proteins	85.3 cal
Carbohydrates	Total Carbohydrates	146 g
	Dietary Fibre	17 g
Fatty acids	Total Fat	8.4 g
	Saturated Fat	1.4 g
	Monounsaturated Fatty Acid	1.5 g
	Polyunsaturated Fatty Acid	4.3 g
	Omega-3 Fatty Acids	236 mg
	Omega-6-Fatty Acids	4 g
Vitamins	Vitamin E	100 µg
	Vitamin K	1.8 µg
	Thiamine	842 µg
	Riboflavin	580 µg
	Niacin	9.4 mg
	Vitamin B6	768 µg
	Foliate	170 µg
	Pantothenic Acid	170 µg
Minerals	Calcium	16 mg
	Iron	6 mg
	Magnesium	228 mg
	Phosphorus	570 mg
	Potassium	390 mg
	Sodium	10 mg
	Zinc	3.4 mg
	Copper	1.5 mg
	Manganese	3.3 mg
	Selenium	5.4 µg
Amino Acids (g/100 g protein)	Leucine	10.7
	Isoleucine	4.4
	Valine	4.9
	Threonine	4.0
	Arginine	4.6
	Lysine	3.1
	Methionine	1.1
	Cisteine	1.5
	Tryptophan	1.4
	Glutamic Acid	23.0
	Alanine	8.7
	Proline	5.8

## Data Availability

Data is contained within the article.
